# Fraxin Promotes the Activation of Nrf2/ARE Pathway via Increasing the Expression of Connexin43 to Ameliorate Diabetic Renal Fibrosis

**DOI:** 10.3389/fphar.2022.853383

**Published:** 2022-03-24

**Authors:** Rui Chen, Jingran Zeng, Chuting Li, Haiming Xiao, Shanshan Li, Zeyuan Lin, Kaipeng Huang, Juan Shen, Heqing Huang

**Affiliations:** ^1^ Laboratory of Pharmacology and Toxicology, School of Pharmaceutical Sciences, Sun Yat-Sen University, Guangzhou, China; ^2^ Phase I Clinical Trial Center, Guangzhou Eighth People’s Hospital, Guangzhou Medical University, Guangzhou, China; ^3^ Guangdong Provincial Key Laboratory of Pharmaceutical Bioactive Substances, Guangdong Pharmaceutical University, Guangzhou, China

**Keywords:** fraxin, diabetic nephropathy, oxidative stress, Connecxin43, AKT, Nrf2/ARE pathway

## Abstract

Diabetic nephropathy (DN) is quickly becoming the largest cause of end-stage renal disease (ESRD) in diabetic patients, as well as a major source of morbidity and mortality. Our previous studies indicated that the activation of Nrf2/ARE pathway via Connexin43 (Cx43) considerably contribute to the prevention of oxidative stress in the procession of DN. Fraxin (Fr), the main active glycoside of Fraxinus rhynchophylla Hance, has been demonstrated to possess many potential pharmacological activities. Whereas, whether Fr could alleviate renal fibrosis through regulating Cx43 and consequently facilitating the activation of Nrf2/ARE pathway needs further investigation. The *in vitro* results showed that: 1) Fr increased the expression of antioxidant enzymes including SOD1 and HO-1 to inhibit high glucose (HG)-induced fibronectin (FN) and inflammatory cell adhesion molecule (ICAM-1) overexpression; 2) Fr exerted antioxidant effect through activating the Nrf2/ARE pathway; 3) Fr significantly up-regulated the expression of Cx43 in HG-induced glomerular mesangial cells (GMCs), while the knock down of Cx43 largely impaired the activation of Nrf2/ARE pathway induced by Fr; 4) Fr promoted the activation of Nrf2/ARE pathway via regulating the interaction between Cx43 and AKT. Moreover, in accordance with the results *in vitro*, elevated levels of Cx43, phosphorylated-AKT, Nrf2 and downstream antioxidant enzymes related to Nrf2 were observed in the kidneys of Fr-treated group compared with model group. Importantly, Fr significantly improved renal dysfunction pathological changes of renal fibrosis in diabetic db/db mice. Collectively, Fr could increase the Cx43-AKT-Nrf2/ARE pathway activation to postpone the diabetic renal fibrosis and the up-regulation of Cx43 is probably a novel mechanism in this process.

## Introduction

Diabetic nephropathy (DN) is one of the serious chronic microvascular complications of diabetes mellitus, which is rapidly becoming the major cause of end-stage renal disease (Tervaert et al., 2010; Kanwar et al., 2011). Renal fibrosis is the main pathological feature of DN, which is characterized by glomerulosclerosis and tubulointerstitial fibrosis (Kato and Natarajan, 2014). Glomerular mesangial cells (GMCs) are the main functional cells of the glomerulus (Qian et al., 2008). The overproduction of inflammatory cell adhesion molecule (ICAM-1) and the progressive accumulation of extracellular matrix (ECM) such as fibronectin (FN)in GMCs are the main causes of renal fibrosis (Chen et al., 2003; Qian et al., 2008). The pathogenesis of DN is complex, and it has gradually become a consensus that chronic oxidative stress induced by multiple factors is the main link that leads to diabetic renal fibrosis (Schena and Gesualdo, 2005; Ozbek, 2012; Jha et al., 2016). Increasing evidence indicates that the nuclear factor-erythroid 2-related factor 2 (Nrf2) is the most important intracellular endogenous antioxidant stress pathway that plays a central role in protecting cells from oxidative or electrophilic stress (Yu and Kensler, 2005; Lu et al., 2016). The activation of Nrf2 has been shown to inhibit the excessive accumulation of intracellular reactive oxygen species (ROS) by regulating the expression of endogenous antioxidant enzymes, which in turn inhibits the development of oxidative stress-induced fibrotic injury in DN (Li et al., 2012; Bhakkiyalakshmi et al., 2015; Matzinger et al., 2018). Therefore, promoting the activation of Nrf2/ARE signaling pathway to alleviate renal oxidative stress would be an effective therapeutic strategy to ameliorate renal fibrosis.

Gap Junctions (GJ) are channels between adjacent cells consisting of connexins (Cxs) subunits, which can interact with various of signaling proteins and scaffold proteins through intercellular gap communication and its carboxyl-terminal (CT) independent of communication function to regulate cell functions (Hervé et al., 2004; Harris, 2007). Cx43 is one of the most widely expressed proteins in the Cxs family, and studies have confirmed that Cx43 has a high expression in the kidney (Prakoura et al., 2018). Recently, attention has focused on the negative regulation of oxidative stress by Cx43 during several disease processes. Notably, a significant reduction in Cx43 expression was observed in the diabetic kidneys, which has been confirmed to response for the oxidative stress in kidneys (Xie et al., 2013; Guo et al., 2014; Sun et al., 2021). Additionally, our previous study showed that the up-regulation of Cx43 could inhibit oxidative stress and reduce the overexpression of FN and ICAM-1 in GMCs induced by high glucose (HG) *via* activating the Nrf2 pathway (Chen et al., 2017). Recently, serine/threonine kinase (B), also known as AKT, has been confirmed to interact with the carboxyl terminus of Cx43 and mediate Cx43 signaling cascade.(Hervé et al., 2007; Leithe et al., 2018). Inhibition of AKT protein phosphorylation was associated with an enhanced renal inflammatory response and renal cell death in the DN model (Welsh et al., 2005; Lou et al., 2020). Furthermore, the activation of AKT was confirmed to facilitate the nuclear translocation of Nrf2 and contribute to the positive regulation of antioxidation in multiple clinical investigations (Huang et al., 2018; Bai et al., 2019). Therefore, Cx43’s carboxyl terminus interacted with AKT and consequently promoted the activation of the Nrf2 signaling pathway, which is probably a novel mechanism for Cx43 to inhibit oxidative stress and ameliorate diabetic kidney fibrosis.

Fraxin (Fr) is the main active component of Fraxinus rhynchophylla Hance and has various pharmacological and biological activities (Kostova, 2001). Esculin, a structural analog of Fr, was found to reduce blood glucose in streptozotocin (STZ)-induced diabetic mice by inhibiting inflammatory processes associated with oxidative stress and also suppress the development of diabetic renal dysfunction (Kang et al., 2014). Another research showed Fr could reduce renal damage in ischemia-reperfusion injured kidneys by inhibiting oxidative stress and anti-inflammatory pathways (Topdağı et al., 2020). Given that antioxidant treatment is beneficial for the improvement of renal fibrosis, there is a pressing need to better elucidate whether the antioxidant effect of Fr contributes to its protective activity during DN. In this study, we demonstrated that Fr was effective in increasing antioxidant enzyme and improving renal function in glomeruli in diabetic db/db mice. Further studies have found that Fr could upregulate the expression of Cx43 protein as well as facilitate the activation of Nrf2/ARE signaling pathway. Moreover, the specific mechanism may be related to the promotion of the interaction of Cx43 with AKT, and thereby promoting the activation of the Nrf2/ARE signaling pathway.

## Materials and Methods

### Reagents and Antibodies

Penicillin and streptomycin (catalogue: V900929) were purchased from Life Technologies ™ (Grand Island, NY, United States). Dulbecco’s modified Eagle’s medium (DEME, catalogue: 31,600-034), fetal bovine serum (FBS, catalogue: 10,270-106), Lucifer yellow (catalogue: l453), RNAiMAX (catalogue: 13778030) and Lipofectamine^®^ LTX and Plus reagents (catalogue:15338100) were purchased from Life Technologies ™ (Grand Island, NY, United States). d-Glucose (catalogue: 0188) was purchased from Amresco (Solon, OH, United States). Fr for animal experiments was purchased from Nanjing Diger Medical Technology (catalogue: D106523-10g; purity >98.0%, HPLC; Nanjing, China), and Fr for cells was purchased from Ronghe (purity >99.0%, HPLC; Shanghai, China). Nuclear Extract Kit was purchased from Active Motif (Carlsbad, CA, United States). Total SOD (catalogue: S0101) Assay Kit with WST-8, Lipid Peroxidation MDA (catalogue: S0131) Assay Kit and hydrogen peroxide detection kit (catalogue: S0038) were purchased from Beyotime (Haimen, China).

Antibodies against α-Tubulin (catalogue: 66031-1-Ig), FN (catalogue: 15613-1-Ap), ICAM-1 (catalogue: 10020-1-Ap), Nrf2 (catalogue: 16396-1-Ap), HO-1 (catalogue: 10701-1-Ap), SOD1 (catalogue: 10269-1-Ap), p-Akt (catalogue: 66444-1-Ig) and Akt (catalogue: 10176-2-Ap) were purchased from Proteintech group (Chicago, IL, United States). Antibodies against Lamin B1 (catalogue: ab133741) was purchased from Abcam (Cambridge, Ma, United States). Connexin 43 (catalogue: 3512) was purchased from Cell Signaling Technology (Boston, Ma, United States). TBHQ (catalogue: HY-100489) and MK-2206 (catalogue: HY-10358) were purchased from MedChem Express (New Jersey, United States). Mouse IgG (catalogue: A0216) and Methylthiazoly ldiphenyl-tetrazolium bromide (MTT, catalogue: ST316) were obtained from Beyotime (Haimen, China). The Dual-Glo luciferase Assay System (catalogue: E2920) was purchased from Promega Corporation (Madison, WI, United States). Horseradish peroxidase conjugated secondary antibody was purchased from Promega Corporation (Madison, WI, United States). Alexa Fluor^®^ 488 Goat anti-rabbit IgG and Goat anti-rabbit IgG labeled with Alexa fluor^®^ 594 were purchased from Thermo Fisher Scientific (Rockford, IL, United States).

### Cell Culture

Primary GMCs were isolated from the glomerular cortex of male Sprague Dawley (SD) rats (150–180 g) by using the same method as previously described (Geoffroy et al., 2004). Briefly, the cortical part was cut and mechanically passed through 230, 73.7 and 70 μm stainless steel screens subsequently, and the glomeruli were digested with 0.1% type IV collagenase. Finally, cells were cultured with DMEM medium containing 20% fetal bovine serum, 0.66 U/ml insulin, 2 mM glutamine, 100 U/ml penicillin and 100 U/ml streptomycin in a 5% CO_2_ incubator at 37 C. The culture medium was changed for the first time after 4 days and every 2–3 days thereafter when the GMCs grew to the bottom of the bottle. GMCs from generation 5 to 13 were used for the subsequent experiments, and were cultured in DMEM containing 10% fetal bovine serum at 37 C in a 5% CO_2_ incubator. Before treatment, GMCs were serum-starved for 16 h and then treated with glucose (5.6 mM for normal glucose and 30 mM for HG).

### MTT Assay

GMCs were inoculated in 96-well plates, and 100 μl of DMEM low-sugar medium was added to each well. 24 h later, cells were treated with different concentrations of Fr (10, 20, 40, 60, 80, 160, 320 μM) for 24 h. Then 20 μL of MTT (3-(4, 5-dimethylthiazol-2-yl)-2, 5-diphenyl tetrazolium bromide, 5 mg/ml, sigma, United States) was added to each well, resulting in a final MTT concentration as 0.5 mg/ml, and the plate was incubated at 37 C for 4 h. To dissolve the formazan crystals, 200 μl of DMSO was added to each well after the medium was thoroughly discarded. The cell viability was then calculated using an enzyme-linked immunoassay that detected absorbance at 490 nm wavelength.

### Western Blot Assay

The western blot assay was carried out consistent with the previously described steps (Xiao et al., 2020). Proteins of kidney tissue and GMCs were extracted by using RIPA lysis buffer with protease inhibitor cocktail, phosphatase inhibitor A and B for 30 min. Commercial kits were used for nuclear and cytoplasmic protein extraction. Protein concentrations were determined using BCA protein assay kits and equal amounts of proteins were subsequently separated from cells or tissues by 8 %–12% sodium dodecyl sulfate polyacrylamide gel electrophoresis (SDS-PAGE). And then the gels were transferred to PVDF membranes (Bio Rad, Hercules, CA, United States). Non-specific binding was blocked with 5% milk for 1 h at room temperature. After washing with 0.01% Tween-20/TBS (TBST), the membrane was incubated with primary antibodies for more than 12 h at 4 C. Finally, the blots were visualized by using GE Image Quant LAS4000mini (GE healthcare, Waukesha, WI, United States), which was followed by using the ImageJ protein analysis software to perform protein gray analysis (Bio Rad, Hercules, CA, United States) (Version 4.6.2).

### Small-Interfering RNAs and Transient Transfection

Small-interfering RNA (siRNA) of Cx43 was purchased from GenePharma (GenePharma, shanghai, China). The sequences of Cx43-siRNA were as follows:

sense: 5′-CCU​UGG​UGU​CUC​UCU​CGC​UUU​TT-3′

antisense: 5′-AAA​GCG​AGA​GAC​ACC​AAG​GTT-3′

GMCs were placed in 35 mm plates before transfection for 24 h, and then 5 μl of siRNA (50 nM) and 5 μl of Lipofectamine^®^ RNAiMax Reagent (1:1) were transfected into cells according to the protocol. After serum-free for more than 12 h, the cells were processed for the next step.

### Immunofluorescence Staining

GMCs were seeded and cultured on glass coverslips in 24-well plates to 60 %–70% confluence and then were treated with various stimuli. After treatment, the cells were washed 3 times with PBS, then were fixed with 4% paraformaldehyde at room temperature for 15 min. Then the cell membrane was broken with 1% Triton X-100 at room temperature for 10 min. The plates were blocked with 10% goat serum at room temperature for 1 h. Finally, the cells were incubated with Nrf2 primary antibody (diluted with 1:200) overnight at 4 C. After washing with PBS, Alexa fluor 488 conjugated secondary antibody was incubated for 1 h at room temperature in the dark, and then incubated with DAPI dihydrochloride (5 mg/ml in PBS, Sigma, St. Louis, Mo) for 10 min at room temperature in the dark. Finally, the fluorescence quencher (Beyotime, Haimen, China) was added and the slides were sealed on slides. The images were captured by using a Zeiss LSM 510 laser confocal fluorescence microscope (Carl Zeiss, Oberkochen, Germany).

### Detection of Intracellular Superoxide and H_2_O_2_ Levels

Cells were seeded in 96-well plates at appropriate dilution ratios and treated with indicated stimulation. The fluorescent probe dihydroethidium (DHE, Beyotime, Haimen, China) was diluted at a 1:1000 ratio and added to each well. After incubating at 37 C for 30 min, the plate was washed three times with PBS. The fluorescence was measured utilizing high content screening.

The H_2_O_2_ level in GMCs was measured by using a hydrogen peroxide assay kit (Beyotime, Haimen, China). Firstly, the lysis solution provided by the kit was added to the cells at the recommended ratio according to the instructions, and then the cell suspension was collected and broken by sonication. After incubating on ice for 30 min, the supernatant was collected by centrifugation at 1000 rpm for 3 min for further experiments. Finally, 50 μl supernatant and 100 μl assay solution were added to a 96-well plate, and measured absorbance at 560 nm with a microplate reader (Omerga, Norwalk, CT, United States) after standing for 30 min at room temperature. The concentration of H_2_O_2_ released was calculated according to the standard concentration curve derived from the standard solution.

### Dual Luciferase Reporter Assay

The DNA transcriptional activity of Nrf2 was measured by dual luciferase reporter assay according to the manufacturers’ instructions. Briefly, GMCs were seeded in 96-well plates and incubated at 37°C for 24 h before starting transient transfection, and then 0.2 μg of pARE-Luc reporter gene plasmid (Beyotime) and 0.04 μg of pRL-TK (Promega, Madison, WI, United States) were co-transfected. After transfection for 24 h, cells were treated with indicted stimulations, and then the cells were collected according to the Dual-Luciferase reporter gene assay kit (Promega) and the effect of Fr on the transcriptional activity of the Nrf2 pathway was analyzed using the Dual-Luciferase^®^ reporter assay system kit (Promega).

### Immunoprecipitation Assay

GMCs were lysed on ice for 30 min with IP lysis solution, and then the supernatants were collected by centrifugation at 12,000 g for 10 min for BCA protein quantification. 400 μg proteins were incubated with 2 μg Cx43 antibody or negative control antibody IgG overnight at 4°C. Prior to immunoprecipitation, 400 μg whole cell lysates were incubated with 15 μl protein agarose A/G beads (Pierce, Rockford, IL, United States) for 3–4 h to reduce non-specific combinations, and then centrifuged at 12,000 g for 30 s, discarded the supernatants and washed the sediment three times with IP Buffer. After adding 25 μl SDS-PAGE loading buffer and boiled for 5 min, the immunoprecipitation was separated by western blot, and then analyzed by western blot with the respective antibodies.

### Animal Experiment

The animal experiment was approved by the Ethics Committee for the Care and Use of Laboratory Animals of Sun Yat-Sen University (Guangzhou, China). 5-week-old male specific pathogen-free (SPF)-grade db/db mice (n = 50) and db/m (n = 10) mice from Jiangsu Collective Pharmachem Biotech Company. All animals were housed under specific sterile conditions, and all experimental procedures were strictly performed strictly to the standards of the Guide for the Care and Use of Laboratory Animals published by the National Institutes of Health.

After the animals were adaptively fed for 1 week, mice were randomly divided into six groups according to blood glucose values: normal group (n = 10), model group (n = 10), the low dose group of Fr (25 mg/kg, n = 10), the medium dose group of Fr (50 mg/kg, n = 10), the high dose group of Fr (100 mg/kg, n = 10), and the positive drug group of valsartan (Val) (10 mg/kg, n = 10). The mice was treated administration with Fr or valsartan for 8 weeks, 6 days per week, in a volume of 0.1 ml/10 g. The normal group of mice was treated with a dose of CMC-Na at a dose of 0.1 ml/10 g as a blank control. All animals were housed in specific pathogen-free (SPF) conditions in a temperature-controlled (20C–25 C) and humidity-controlled (40 %–70%) barrier system with a 12 h light and dark cycle. And during the last week of the animal experiments, 24 h urine was collected from the mice using metabolic cages, and the volume and status of the urine was observed and recorded. At the end of the experiment, the blood was collected in anti-coagulation tubes with sodium heparin, let it stand still for 30 min at room temperature for 30 min, and then was centrifuged at 4°C, 3000 rpm for 10 min. And the supernatant was collected for subsequent biochemical index testing. The kidney tissues of mice were stored at −80°C for subsequent experiments.

### Biochemical Analysis

Glycated serum protein (GSP), glycated blood red (HbAlc), blood urea nitrogen (BUN), serum creatinine (Cr) and 24 h urine protein (UP) levels were measured by using commercially available kits (Jiancheng Biotech, Nanjing, China). In addition, malondialdehyde (MDA) levels and total superoxide dismutase (SOD) activity in serum and kidney tissues were measured using commercially available kits (Beyotime, Haimen, China). Kidney tissues were fixed with 4% paraformaldehyde and paraffin embedded, and then kidney sections (4 μm thick) were applied for PAS, HE and Masson staining. The images were photographed by light microscopy and the images were quantitatively analyzed.

### Data and Statistical Analysis

The experimental data were expressed as mean ± SEM (Mean ± SEM), and the Graphpad Prism 5.0 statistical software was used for all statistical analysis. *t*-test was used for comparison between two groups and the comparison between multiple groups were analyzed by one-way analysis of variance (one-way ANOVA, Bonferroni method), *p* < 0.05 is considered as the difference is statistically significant.

## Results

### Fr Suppressed the Development of Inflammatory Fibrosis Factors by Boosting the Expression of Antioxidant Enzymes in High Glucose Induced GMCs

**FIGURE 1:**
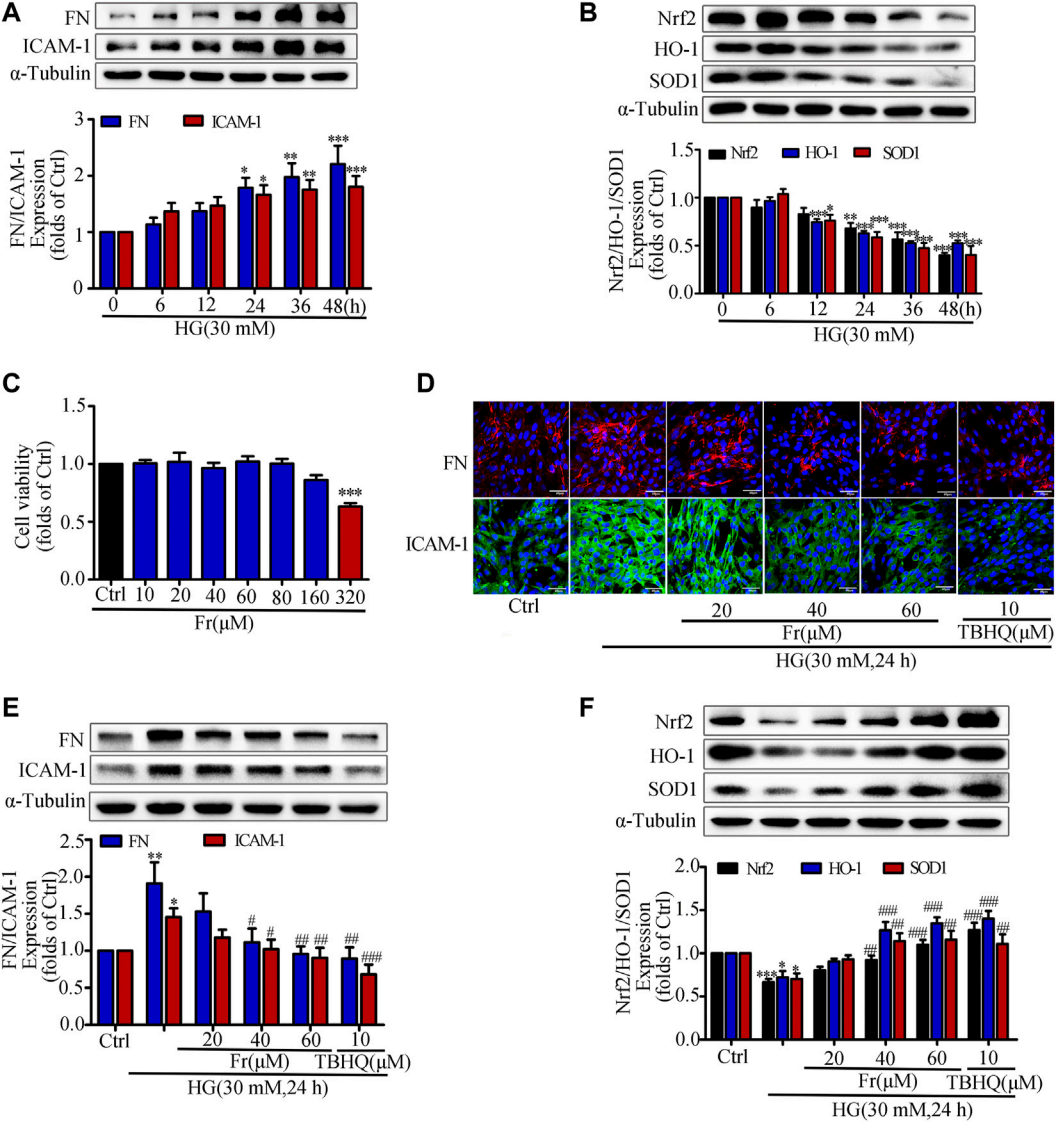
Fr suppressed the development of inflammatory fibrosis factors by boosting the expression of antioxidant enzymes in high glucose induced GMCs. **(A-B)** The protein expression of FN, ICAM-1, Nrf2, HO-1 and SOD1 in GMCs were measured by western blot assay, ^*^ *p* < 0.05, ^**^ *p* < 0.01, ^***^ *p* < 0.001 *vs*. 0 h. **(C)** Cell survival analysis through MTT assay of GMCs was treated with different concentrations of Fr for 24 h, ^***^ *p* < 0.001 *vs*. Ctrl. **(D)** The cellular distribution of FN and ICAM-1 was untreated or treated through Immunofluorescence (magnification ×400). Bar: 40 μm. The red and green fluorescence represent the localization of FN and ICAM-1 respectively. **(E-F)** The protein levels of FN, ICAM-1, Nrf2, HO-1 and SOD1 in GMCs were measured through western blot assay, ^*^ *p* < 0.05, ^**^ *p* < 0.01, ^***^ *p* < 0.001 *vs*. Ctrl; ^#^ *p* < 0.05, ^##^ *p* < 0.01, ^###^ *p* < 0.001 *vs*. HG. Independent experiments were performed at least three times with similar results.

Previous studies have shown that hyperglycemia induces GMCs to overproduce ECM such as FN, thereby promoting glomerulosclerosis in the diabetic state (Schena and Gesualdo, 2005; Miller et al., 2014; Shotorbani et al., 2020). In addition, the excessive secretion of inflammatory adhesion factors such as ICAM-1 caused by multiple factors is one of the main reasons for the progressive accumulation of renal ECM (Elmarakby et al., 2011; Wu et al., 2019). The kidney is particularly susceptible to damages caused by increased oxidative stress, and numerous studies have indicated that the Nrf2/ARE pathway is an important antioxidative stress protective system (Gnudi et al., 2007; Sagoo and Gnudi, 2018; Li et al., 2021). After HG treatment of GMCs at different time points (0, 6, 12, 24, 36, and 48 h), we found that HG could significantly increase the expression of FN and ICAM-1 (Figure 1A), and reduce the expression of Nrf2, HO-1 and SOD1 (Figure 1B). HG stimulation for 24 h triggered significant upregulation of these inflammatory fibrotic factors compared with the control group. Therefore, we used 30 mM HG to stimulate GMCs for 24 h as the modeling condition in the subsequent experiments. The MTT findings revealed that Fr concentrations less than 320 μM had no influence on cell viability (Figure 1C). As a result, we chose 20, 40, and 60 μM Fr for the subsequent studies. After treating HG-induced GMCs with various concentrations of Fr, we found that Fr significantly inhibited the expression of FN and ICAM-1 with the dose-dependent effect (Figures 1D,E), and the inhibitory effect of 60 μM of Fr was comparable to that of 10 μM of the Nrf2 specific agonist TBHQ. In addition, HG induction decreased the expression of Nrf2, HO-1 and SOD1 in GMCs, while administration of Fr reversed (Figure 1F). All data above indicated that Fr could inhibit the production of inflammatory fibrotic components in HG-induced GMCs and promote the activation of Nrf2 signaling pathway.

### Fr Inhibited Oxidative Stress by Activating the Nrf2/ARE Pathway in HG-Induced GMCs

**FIGURE 2:**
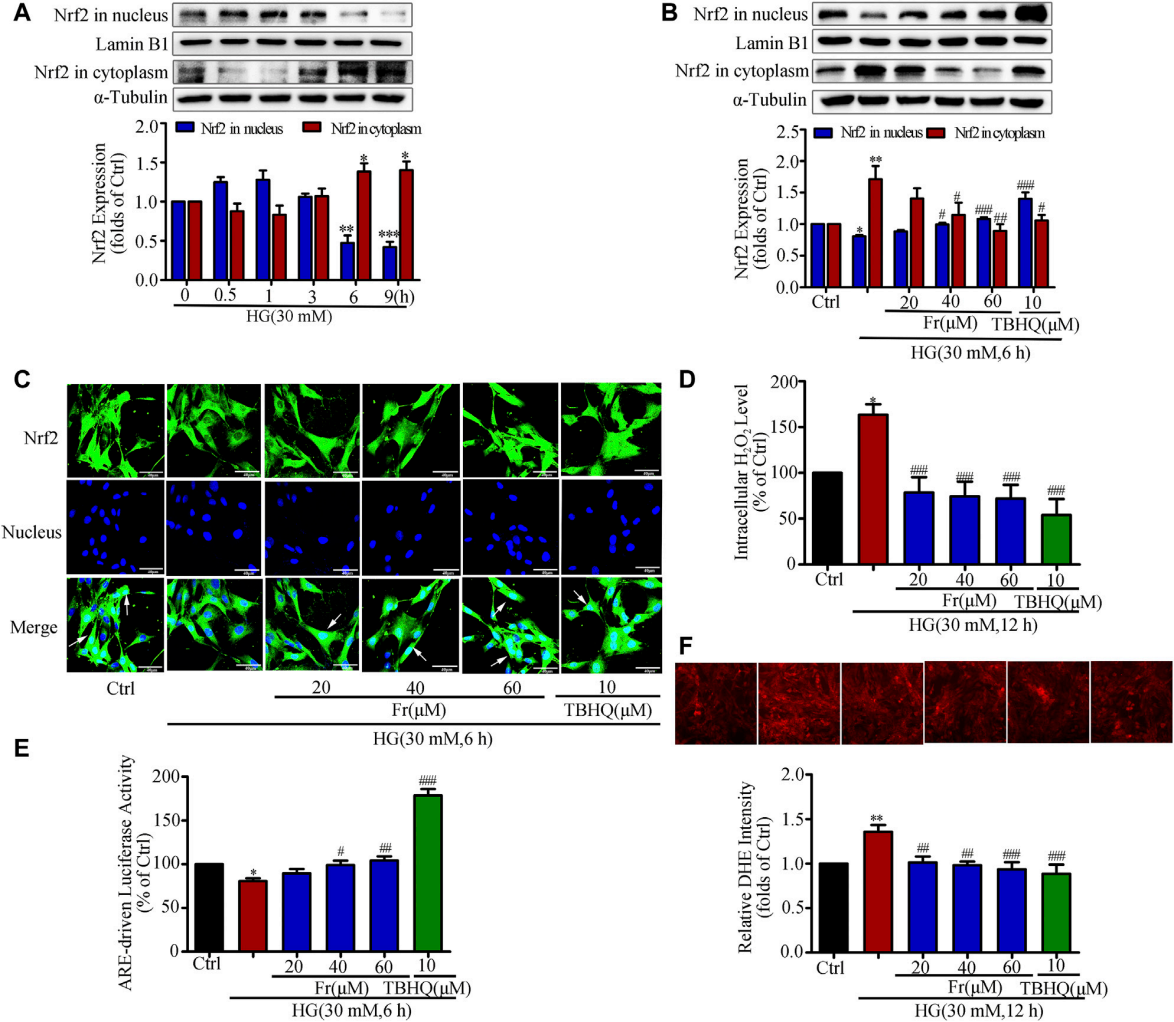
Fr inhibited oxidative stress by activating the Nrf2/ARE pathway in HG-induced GMCs. **(A)** The western blot results showed the expression of Nrf2 in the nucleus and cytoplasm. ^*^ *p* < 0.05, ^**^ *p* < 0.01, ^***^ *p* < 0.001 *vs*. 0 h. **(B)** Western blot analysis of nuclear and cytoplasm Nrf2 in GMCs treated with HG and Fr for 24 h ^*^ *p* < 0.05, ^**^ *p* < 0.01 *vs*. Ctrl; ^#^ *p* < 0.05, ^##^ *p* < 0.01, ^###^ *p* < 0.001 *vs*. HG. **(C)** The subcellular distribution of Nrf2 in GMCs was measured by immunofluorescent staining (×600 magnification). Bar: 40 μm. Green fluorescence indicated localization of Nrf2. **(D)** Intracellular H_2_O_2_ level was provoked by high glucose and Fr treatment for 12 h *p < 0.05 *vs*. Ctrl; ^###^ *p* < 0.001 *vs*. HG. **(E)** Transcriptional activity of Nrf2 was measured by luciferase reporter assay. ^*^ *p* < 0.05 *vs*. Ctrl; ^#^ *p* < 0.05, ^##^ *p* < 0.01, ^###^ *p* < 0.001 *vs*. HG. **(F)** Fluorescent probe DHE was applied to detect the intracellular ROS levels for 12 h ^**^ *p* < 0.01 *vs*. Ctrl; ^#^ *p* < 0.05, ^##^ *p* < 0.01 *vs*. HG. Independent experiments are performed at least three times with similar results.

It is reported that HG stimulation could trigger the oxidative response and increased ROS production (Zhang et al., 2018). The western blot results showed adaptive activation of Nrf2 was found for a short period of time, but HG stimulation for 6 h was able to reduce the Nrf2 expression in the nucleus (Figure 2A). Therefore, the following experimental condition was 30 mM HG stimulation for 6 h. Additionally, both Fr with different dosages and TBHQ were able to promote Nrf2 translocation into the nucleus and the reduce cytoplasmic Nrf2 expression (Figure 2B). Further, the intranuclear distribution of Nrf2 in GMCs was considerably decreased, which was dose-dependently enhanced by Fr in turn (Figure 2C). As demonstrated in our previous investigation, treatment with HG (12 h) resulted in significant increases in ROS and H_2_O_2_ generation in GMCs, and therefore the 12 h time point was chosen (Chen et al., 2017). The results showed that H_2_O_2_ and superoxide levels have risen, whereas these increases were both reduced by Fr treatment (Figures 2D,F). Interestingly, the dual luciferase reporter gene results also revealed that the DNA binding activity of Nrf2 to ARE was enhanced by Fr (Figure 2E). The aforementioned data suggested that Fr could activate the Nrf2/ARE pathway and decrease intracellular oxidative stress and ROS generation.

### Interfering With Cx43 Attenuated High Glucose Induced Activation of the Nrf2/ARE Signaling Pathway in GMCs

**FIGURE 3:**
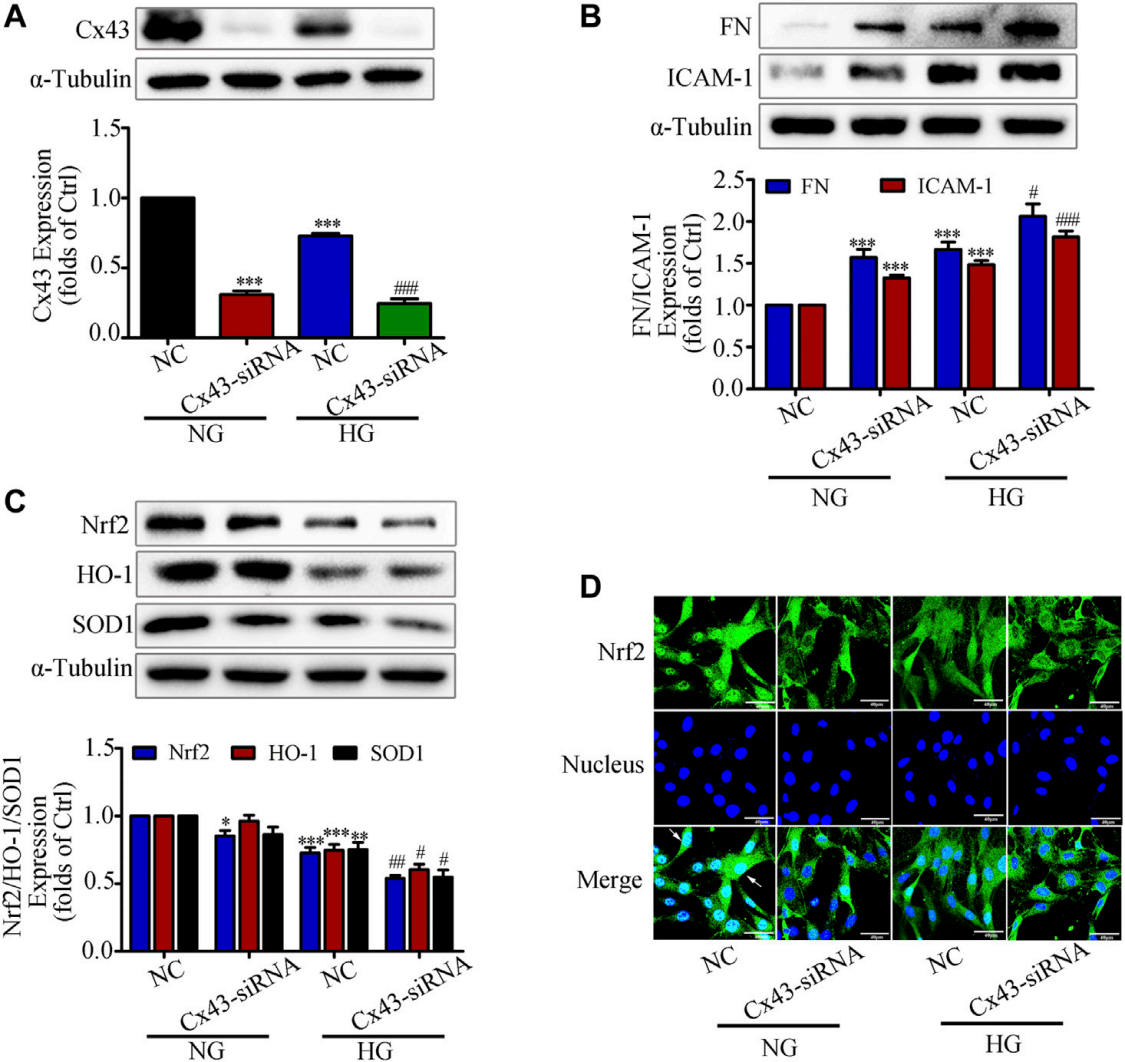
Interfering with Cx43 attenuated high glucose induced activation of the Nrf2/ARE signaling pathway in GMCs. **(A)** The effects of Cx43-siRNA on Cx43 expression in GMCs under 30 mM HG (24 h) conditions was measured by the western blot assay. ^***^ *p* < 0.001 *vs*. (NG + NC); ^###^ *p* < 0.001 *vs*. (HG + NC). **(B-C)** The effects of Cx43-siRNA on the expression of FN, ICAM-1, Nrf2, HO-1, and SOD1was induced by HG (30 mM, 24 h). ^*^ *p* < 0.05, ^**^ *p* < 0.01, ^***^ *p* < 0.001 *vs*. (NG + NC); ^#^ *p* < 0.05, ^##^ *p* < 0.01, ^###^ *p* < 0.001 *vs*. (HG + NC). **(D)** The subcellular distribution of Nrf2 in GMCs was measured by immunofluorescent staining under HG (6 h) conditions (×600 magnification). Bar: 40 μm. Green fluorescence indicated localization of Nrf2. Independent experiments are performed at least three times with similar results.

A recent study reported that overproduction of H_2_O_2_ in Cx43-deficient or Cx43 channel-blocked astrocytes would increase mortality, suggesting that Cx43 expression is closely associated with the onset of oxidative stress (Le et al., 2014). Our previous study also demonstrated that overexpression of Cx43 in GMCs could promote Nrf2/ARE pathway activation to resist excessive ROS production (Chen et al., 2017). Based on the above study background, we investigated whether interfering with Cx43 also had an effect on the activation of Nrf2/ARE pathway. The western blot results indicated that the expression of Cx43 was drastically reduced, suggesting that knockdown of Cx43 was effective (Figure 3A). Furthermore, in the transfected Cx43-siRNA group, there had considerably increased FN and ICAM-1 protein expression, and reduced Nrf2, HO-1, and SOD1 protein expression, as compared to those in the untransfected group (Figures 3B,C). The immunofluorescence data were highly consistent with the immunoblotting results, which further revealed that Nrf2 translocation to the nucleus was significantly reduced by Cx43-siRNA (Figure 3D). The findings provided above revealed that Cx43 had an effect on the activation of the Nrf2/ARE pathway, as evidenced by decreased levels of intracellular oxidative stress and the generation of ROS.

### Fr Promoted Nrf2 Nuclear Translocation by Increasing Cx43 Expression in HG-Induced GMCs

**FIGURE 4:**
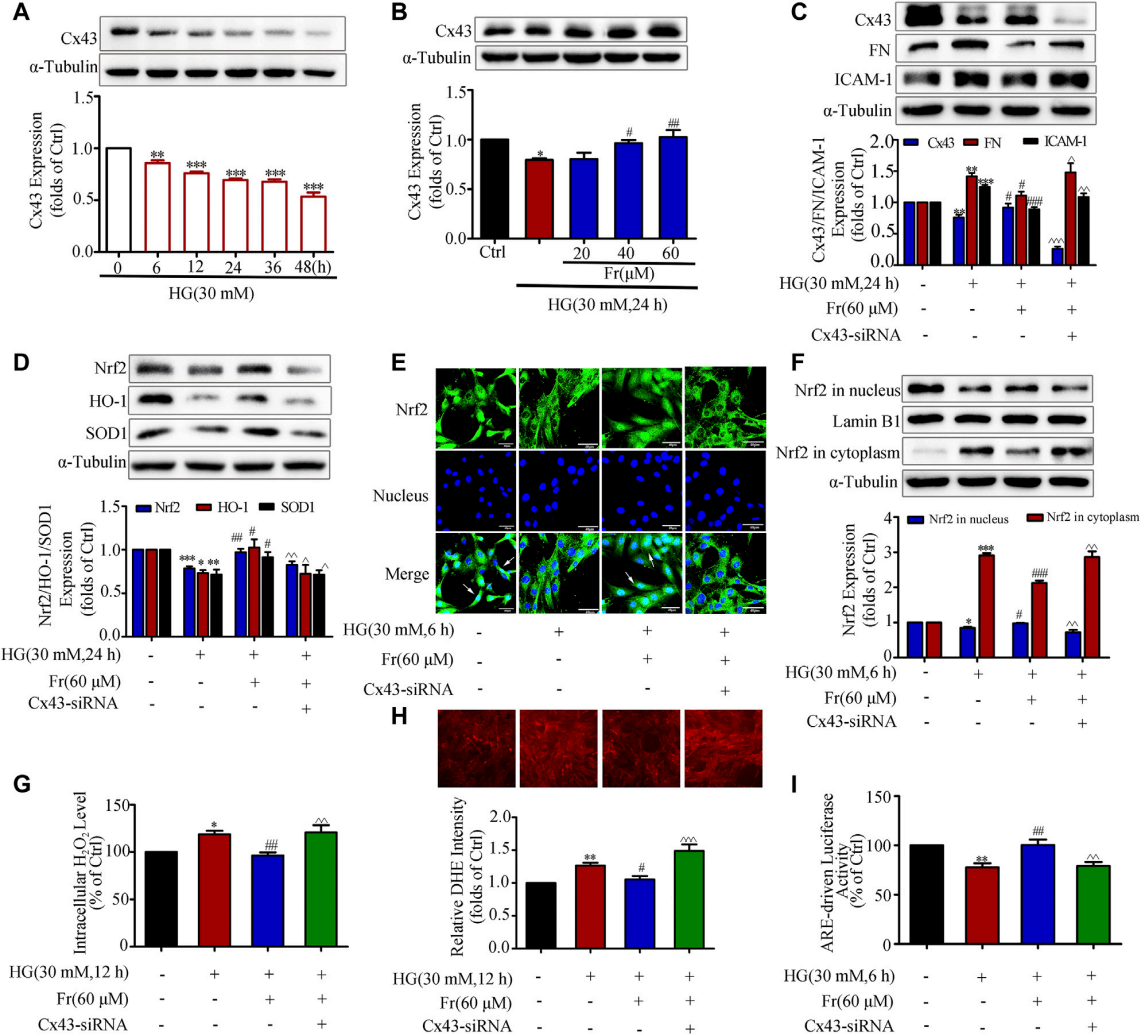
Fr promoted Nrf2 nuclear translocation by increasing Cx43 expression in HG-induced GMCs. **(A-B)** Expression of Cx43 were detected by the western blot assay in GMCs. **(C-D)** The protein levels of Cx43, FN, ICAM-1, Nrf2, HO-1 and SOD1 were detected by the western blot assay. **(E)** The subcellular distribution of Nrf2 in GMCs was measured by immunofluorescent staining (×600 magnification). Bar: 40 μm. Green fluorescence indicated localization of Nrf2. **(F)** The western blot results shown the expression of Nrf2 in the nucleus and cytoplasm. **(G)** The H_2_O_2_ levels in GMCs were detected. **(H)** The intracellular superoxide levels were detected by using the DHE probe. **(I)** Transcriptional activity of Nrf2 was determined by luciferase reporter assay. ^*^ *p* < 0.05, ^**^ *p* < 0.01, ^***^ *p* < 0.001 *vs*. Ctrl; ^#^ *p* < 0.05, ^##^ *p* < 0.01, ^###^ *p* < 0.001 *vs*. HG; ^^^ *p* < 0.05, ^^^^ *p* < 0.01, ^^^^^ *p* < 0.001 *vs*. HG with Fr. Independent experiments are performed at least three times with similar results.

As previous studies demonstrated that overexpression or interference with Cx43 could up-regulate or down-regulate Nrf2 expression (Chen et al., 2017). Then whether Fr could affect the activation of Nrf2/ARE pathway through Cx43 needs to be further clarified. Under the stimulation of HG, intracellular Cx43 expression began to time-dependently decrease from the 6 h (Figure 4A), while Fr could dose-dependently increase intracellular Cx43 protein expression (Figure 4B). To further investigate the mechanism, knockdown of Cx43 was performed. In comparison to Fr administered alone, the results demonstrated that the expression of FN and ICAM-1 was elevated while the Cx43 was lowered after transfection with Cx43-siRNA simultaneously (Figure 4C). Total Nrf2 expression as well as the downstream effector proteins were reduced by Cx43-siRNA (Figure 4D). The results of immunofluorescence also found that the nuclear accumulation of Nrf2 was prevented by the transfection of Cx43-siRNA (Figure 4E), which was further evidenced by immunoblotting (Figure 4F). This was followed by an increase in the formation of intracellular superoxide anion and hydrogen peroxide (Figures 4G,H). The dual luciferase reporter gene experiments also showed that the aggregation of Nrf2 in the nucleus was decreased by Cx43-siRNA compared to cells cultured with Fr alone (Figure 4I).

### Fr Promoted the Activation of Nrf2/ARE Pathway by Regulating the Interaction Between Cx43 and AKT

**FIGURE 5:**
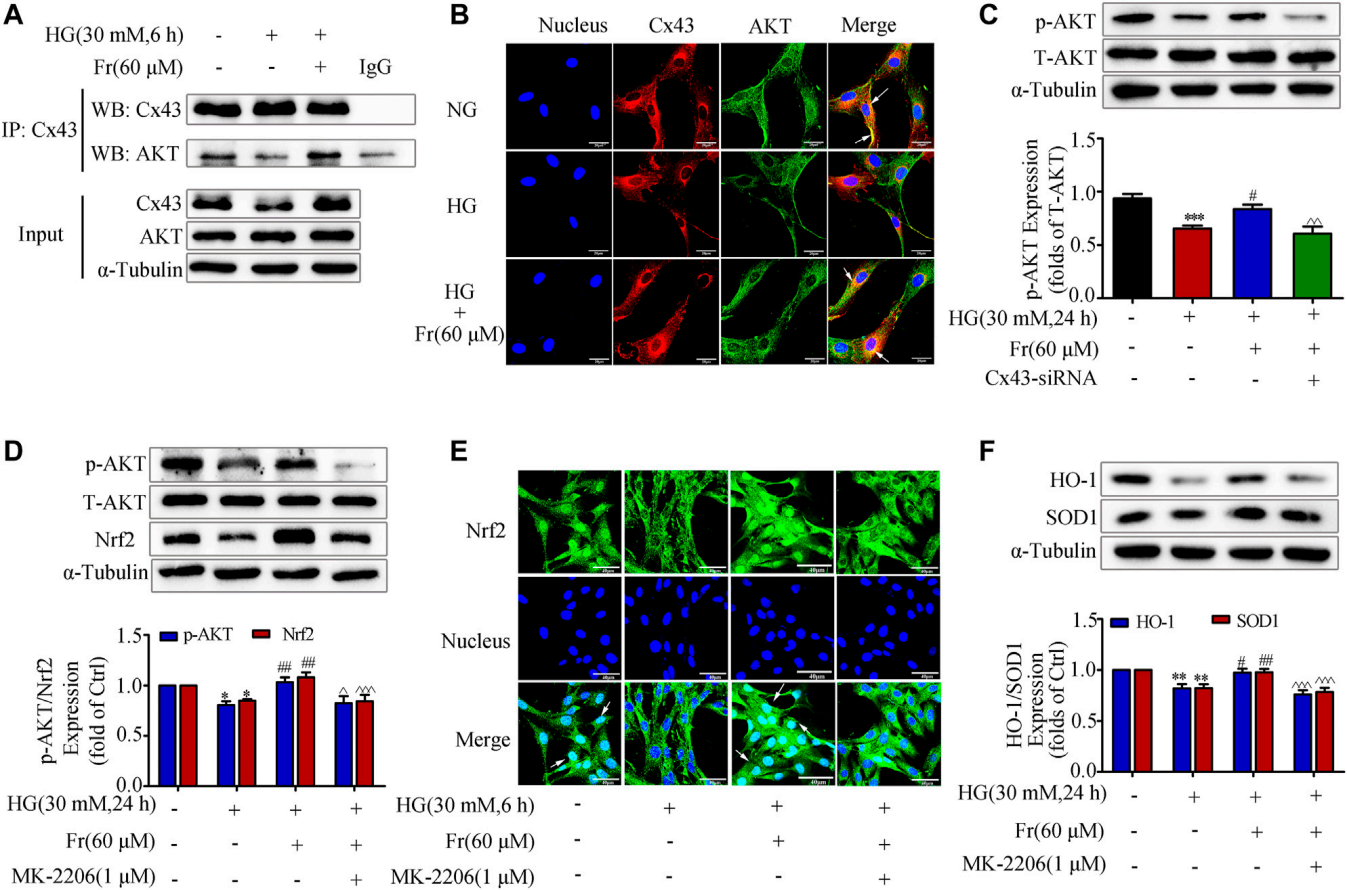
Fr promoted the activation of Nrf2/ARE pathway by regulating the interaction between Cx43 and AKT. **(A)** Immunoprecipitation results of Cx43 suggested that Cx43 interacted with AKT. **(B)** IF results showed that AKT and Cx43 co-localized in the cytoplasm of the GMCs (1000× magnification). Green fluorescence indicated localization of AKT and red fluorescence indicated localization of Cx43. Bar: 20 μm. **(C)** The expression of p-AKT were treated with Cx43-siRNA in GMCs. **(D)** The western blot analysis of the p-AKT and Nrf2in GMCs was treated with MK-2206 and/or Fr. **(E)** The subcellular distribution of Nrf2 in GMCs was measured by immunofluorescent staining (×600 magnification). Bar: 40 μm. Green fluorescence indicated localization of Nrf2. **(F)** The western blot analysis of the HO-1 and SOD1 in GMCs was treated with MK-2206 and/or Fr. ^*^ *p* < 0.05, ^**^ *p* < 0.01, ^***^ *p* < 0.001 *vs*. Ctrl; ^#^ *p* < 0.05, ^##^ *p* < 0.01, ^###^ *p* < 0.001 *vs*. HG; ^^^ *p* < 0.05, ^^^^ *p* < 0.01, ^^^^^ *p* < 0.001 *vs*. HG with Fr. Independent experiments are performed at least three times with similar results.

It is reported that Cx43 could interact with AKT directly via C-terminus in glioblastoma cells (Kuang et al., 2018). Then we speculated whether Cx43 trigger the downstream antioxidant pathway by binding to the AKT, and we further explored the role of AKT as a mediator in this process. The data of IP in Figure 5A showed that Cx43 specifically precipitate AKT under normal conditions, suggesting that Cx43 could interact with AKT. The interaction was further reduced after 6 h of HG treatment, while Fr stimulation increased the interaction (Figure 5A). As shown by the immunofluorescence assay, Cx43 and AKT co-located in the cell membrane and cytoplasm of GMCs. Interestingly, HG treatment could reduce their binding, which was reversed by Fr (Figure 5B). The western blot results revealed that p-AKT protein expression was significantly lowered in the Cx43-siRNA transfection group (Figure 5C). To further explore the important role of AKT in the activation of the Nrf2 signaling pathway induced by Fr, we observed the Nrf2 downstream protein expression after inhibiting the activation of AKT by 1 μM of MK-2206, a phosphorylation inhibitor of AKT. The MK-2206 not only suppressed the expression of AKT phosphorylation, but also declined the up-regulation of total Nrf2 during Fr treatment (Figure 5D), which was accompanied by the decreases of HO-1 and SOD1 levels (Figure 5F). Furthermore, the results of immunofluorescence also confirmed that the nuclear accumulation of Nrf2 was reduced by the AKT phosphorylation inhibitor in HG-induced GMCs (Figure 5E).

### Fr Alleviated Renal Injury and Improved Renal Function in Db/db Mice

**FIGURE 6:**
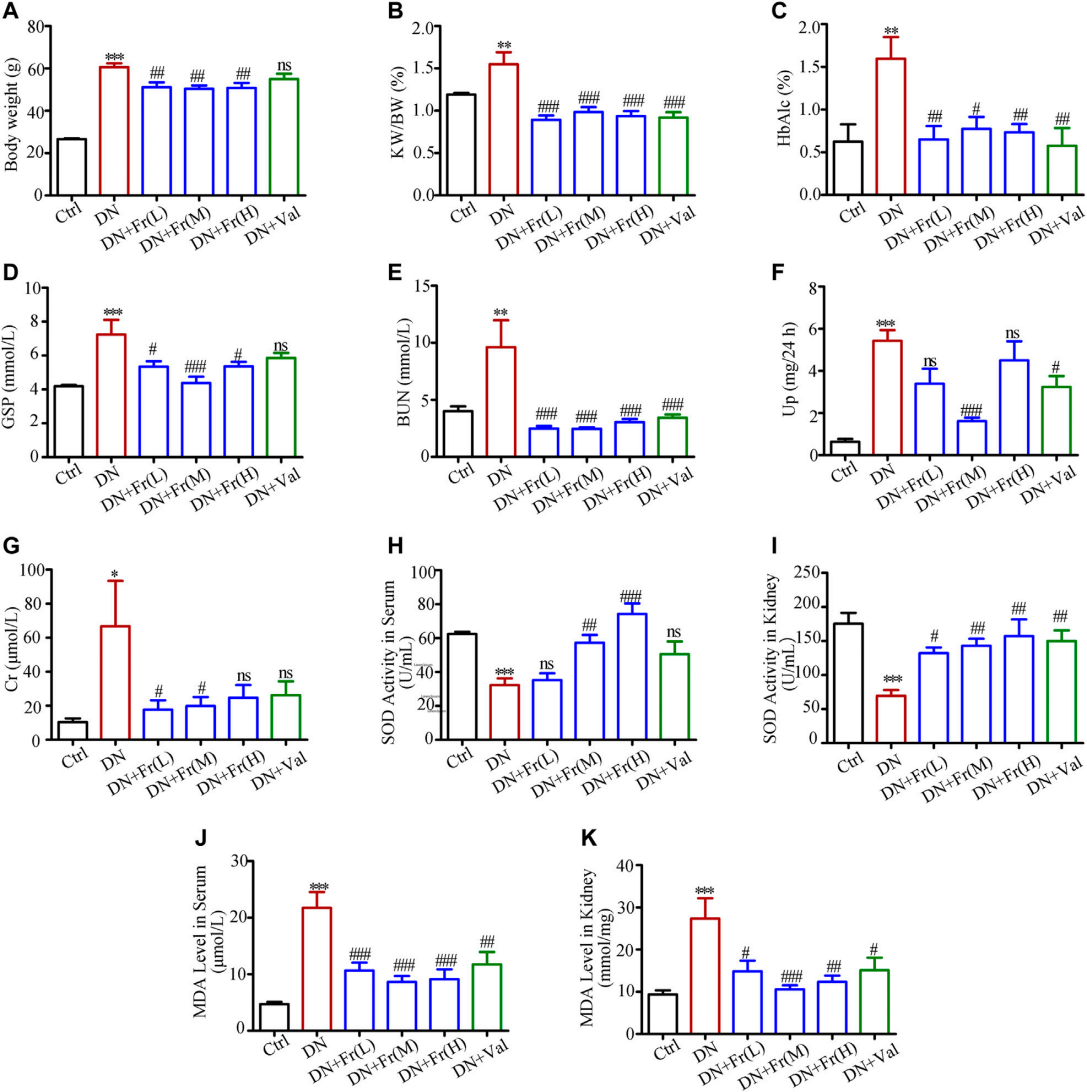
Fr alleviated renal injury and improved renal function in db/db mice. **(A)** Body weight of experimental animals. **(B)** KW/BW, kidney weight/body weight ratio, n = 6 **(C–K)** HbA1c, GSP, BUN, Up, Cr, SOD and MDA of experimental mice. Data were expressed as ±SEM, n = 6, Ctrl: control group; DN: diabetic nephropathy group; DN + Fr (L): Fr treatment group (low dose: 25 mg/kg); DN + Fr (M): Fr treatment group (medium dose: 50 mg/kg); DN + Fr (H): Fr treatment group (high dose: 100 mg/kg); DN + Val: valsartan treatment group (10 mg/kg). ^*^ *p* < 0.05, ^**^ *p* < 0.01, ^***^ *p* < 0.001 *vs*. Ctrl; ns, no significance; ^#^ *p* < 0.05, ^##^ *p* < 0.01, ^###^ *p* < 0.001 *vs*. DN.

The results of *in vitro* experiments have confirmed that Fr could activate the Nrf2/ARE pathway by upregulating the expression of Cx43. Our research group’s preliminary animal investigations indicated that Fr enhanced renal function in diabetic mice in a model of diabetic kidney damage generated by multiple injections of STZ in modest dosages and a high-fat diet. To further analyze the effect of Fr on the renal function of diabetic mice, we applied db/db mice as a diabetic kidney damage model, treated with Fr or Val, and then detected the animals’ blood and urine biochemical indicators. Results showed that Fr or Val could remarkably reverse the abnormal up-regulation of body weight, kidney weight to body weight ratio (KW/BW), glycated hemoglobin (HbAlc), glycated serum protein (GSP), urea nitrogen (BUN), blood creatinine (Cr), and 24 h proteinuria (Up) in db/db mice, which suggested that Fr could improve the renal physiological function of diabetic db/db mice (Figures 6A–G). According to other research, the production of anti-oxidative components such as superoxide dismutase (SOD) in the serum of db/db mice was decreased, although the synthesis of malondialdehyde (MDA) induced by lipid peroxidation was dramatically enhanced (Waldman et al., 2018). Therefore, by detecting SOD activity and MDA levels, we discovered that the SOD activity decreased and the MDA levels increased in the serum and kidney tissues of model mice, whereas the administration of Fr or Val could reverse the changes (Figures 6H–K).

### Fr Reduced the Degree of Renal Fibrosis in Db/db Mice by Inhibiting the Protein Expression of FN and ICAM-1

**FIGURE 7:**
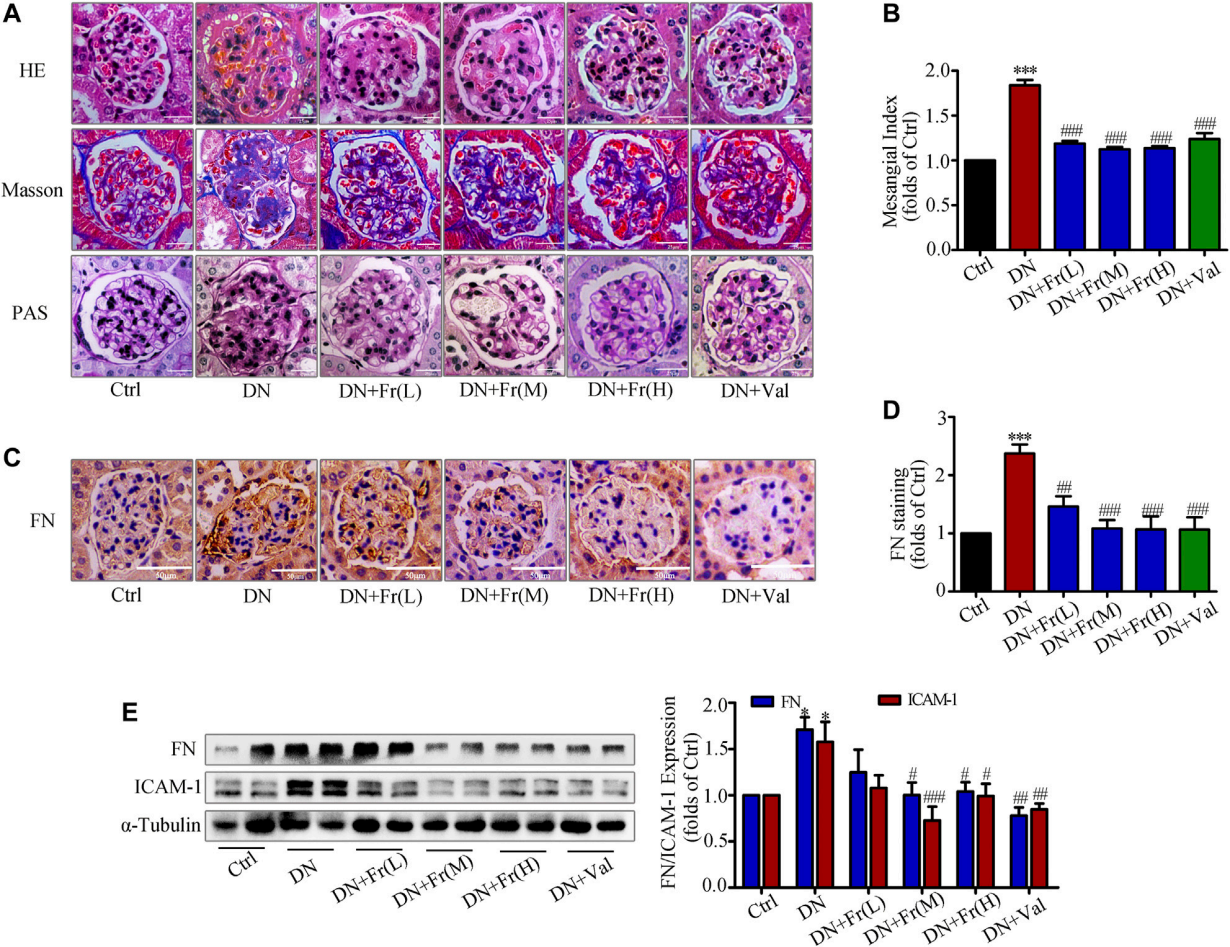
Fr reduced the degree of renal fibrosis in db/db mice by inhibiting the protein expression of FN and ICAM-1. **(A)** Glomerular histopathology analysis was performed by PAS, HE and Masson staining (×400 magnification). Bar: 25 μm, n = 6 **(B)** Statistical analysis of mesangial index. **(C)** The expression of FN in the glomeruli were shown by immunohistochemical staining (×200 magnification). Bar: 50 μm, n = 6. **(D)** Statistical analysis of FN protein expression. **(E)** The levels of FN and ICAM-1 in kidney tissues of db/db mice were detected by the western blot assay. Independent experiments were performed at least three times with similar results. ^*^ *p* < 0.05, ^***^ *p* < 0.001 *vs*. Ctrl; ^#^ *p* < 0.05, ^##^ *p* < 0.01, ^###^ *p* < 0.001 *vs*. DN.

We further observed the effect of Fr on the degree of kidney lesions in db/db mice by staining pathological sections. Hematoxylin and eosin (HE) staining and Masson staining revealed that Fr or Val improved ECM deposition and inflammatory cell infiltration in db/db mice and attenuated excessive collagen fiber deposition in glomeruli (Figure 7A). Periodic acid schiff (PAS) staining showed that the glomeruli of db/db mice were significantly larger and the mesangial matrix was proliferated compared to normal group (Figure 7A). Additionally, administration of Fr or Val reduced glomerular hypertrophy and mesangial zone growth, as well as the mesangial proliferation index (Figure 7B). Immunohistochemistry revealed that FN was elevated in the glomeruli of db/db mice, and all dosage groups of Fr, as well as Val, decreased the expression and distribution of FN in the extracellular matrix (Figures 7C,D). Extraction of proteins from kidney tissues was used in western blot assay, with the result of FN supported immunohistochemistry findings. The protein expression of FN and ICAM-1, both markers of inflammatory fibrosis in kidney tissues, was increased in db/db mice, which reversed by Fr treatment (Figure 7E).

### Fr Stimulated the Activation of Nrf2/ARE Signaling Pathway Up-Regulating Cx43 Expression in Kidney Tissues

**FIGURE 8:**
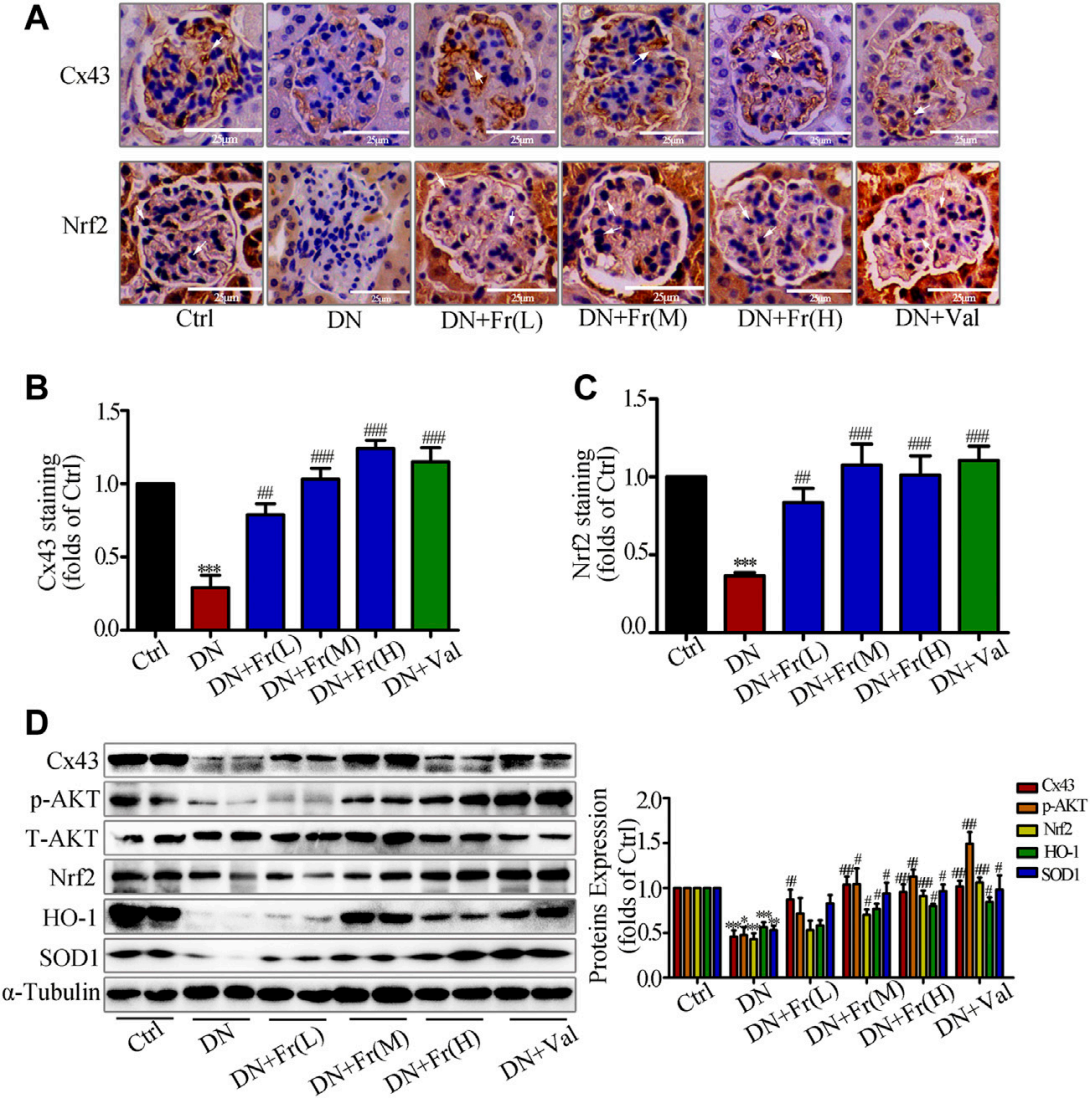
Fr stimulated the activation of Nrf2/ARE signaling pathway by up-regulating Cx43 expression in kidney tissues. **(A)** The expression of Cx43 and Nrf2 in the glomeruli were shown by immunohistochemical staining (×200 magnification). Bar: 50 μm, n = 6. **(B-C)** Statistical analysis of Cx43 and Nrf2 protein expression. **(D)** The levels of Cx43, p-AKT, Nrf2, HO-1, and SOD1 in kidney tissues of db/db mice were detected by the western blot assay. Independent experiments were performed at least three times with similar results. ^*^ *p* < 0.05, ^**^ *p* < 0.01, ^***^ *p* < 0.001 *vs*. Ctrl; ^#^ *p* < 0.05, ^##^ *p* < 0.01, ^###^ *p* < 0.001 *vs.* DN.

On the basis of clarifying the effects of Fr on improving diabetic kidney injury and alleviating the process of renal fibrotic lesions, we examined the effects of Fr on the expression of Cx43 protein and Nrf2/ARE-related proteins in the kidney tissues of db/db mice. Immunohistochemistry showed that the downregulation of Cx43 and Nrf2 expression in glomeruli was reversed by Fr or Val treatment (Figures 8A–C). The Cx43 and p-AKT protein expression was reduced in the kidney tissues of db/db mice compared to normal controls, which was upregulated by Fr treatment (Figure 8D). Furthermore, the expression of Nrf2, SOD1, and HO-1 was also found to be down-regulated, whereas treatment with Fr was able to increase the protein expression levels (Figure 8D). Therefore, these *in vivo* results confirmed that Fr alleviated diabetic renal fibrosis at least partially via up-regulating Cx43 expression and activating the Nrf2/ARE pathway, which ultimately inhibited the renal oxidative damage in diabetic mice.

## Disscusion

DN is an end-stage renal disease caused by long-term hyperglycemia (Xiong and Zhou, 2019). Glomerulopathy, particularly mesangial enlargement and basement membrane thickening, are the most visible pathological alterations in patients with clinical DN (Najafian et al., 2011). GMCs are the main cell type produced ECM and play an important role in maintaining renal tissue structure and physiological function (Tung et al., 2018; Lee et al., 2020). In the development of DN, the overproduction of FN and ICAM-1 in GMCs leads to the renal fibrosis, therefore reducing ECM accumulation in the kidney can prevent glomerulosclerosis and delay the progression of DN (Gorin and Block, 2013). The exact pathogenesis of DN is still unclear, and it is generally believed that oxidative stress, inflammation, and disorders of glucolipid metabolism are related to its occurrence (Arora and Singh, 2013; VR et al., 2019). Excessive ROS generation in GMCs caused by HG can lead to a fall of anti-oxidant enzymes like CAT and SOD, resulting in long-term oxidative stress, as well as facilitate the formation of cytokines and ECM, all of which contribute to the development of diabetic renal fibrosis (Gong et al., 2018). Therefore, reducing the occurrence of oxidative stress is undoubtedly beneficial for the prevention the development of DN. Coumarins have been discovered to improve renal and pancreatic islet structure in diabetic mice via antioxidation, as well as suppress glomerular mesangial matrix synthesis and glomerular basement membrane thickening, suggesting that they might be used as potential hypoglycemic medicines (Yao et al., 2018). Although it is unclear if Fr, a coumarin-like molecule, has similar pharmacological effects. Here, we found that Fr inhibited FN and ICAM-1 expression in HG-induced GMCs via affecting ROS generation, therefore preventing renal fibrosis.

It is evidenced that increased oxidative load leads to the occurrence of diseases including cancer, pulmonary fibrosis and renal fibrosis, and Nrf2 binding to specific ARE promotes the expression of antioxidant enzymes and defense protein genes in a variety of cellular tissues to reduce oxidative load (Walters et al., 2008). We previously discovered that activating the Nrf2 pathway can prevent oxidative stress damage and improve renal function in diabetic renal fibrosis by producing downstream antioxidant proteins (Gong et al., 2017; Zhang et al., 2018). In addition, activation of Nrf2 in renal tubules effectively alleviates tubular injury and interstitial fibrosis by inducing the expression of genes associated with cellular protection against oxidative stress (Nezu and Suzuki, 2020; Potteti et al., 2021). In the present study, we identified that Nrf2 and downstream proteins were also adaptively reduced after treating with HG. Further studies revealed that Nrf2 translocation into the nucleus was also decreased. We subsequently found that Fr enhanced the ARE-binding activity and transcriptional activity of Nrf2. These findings implied that Fr might activate the Nrf2/ARE pathway by promoting the translocation of Nrf2 into the nucleus, reduce oxidative stress in GMCs, and ultimately preventing the formation of renal fibrosis caused by inflammatory factors and ROS.

Cxs play an integral role in the biochemical alterations and pathophysiological disorders of DN (Hanner et al., 2010). In the development of DN, impaired glomerular Cxs function is closely associated with enhanced phosphorylation of Cxs and reduced production of Cxs (Takenaka et al., 2011). The investigators early detected by immunofluorescence that Cx43 is localized between all renal tubular epithelial cells, glomeruli and connective tissue (Beyer et al., 1989). Ball et al. showed that gap junctional intercellular communication (GJIC) activity was decreased in STZ-induced diabetic rat brain astrocytes and hypothalamus slices, which was associated with a decrease in Cx43 expression (Ball et al., 2011). Moreover, our previous study found that Cx43 could inhibit the up-regulation of fibrosis-related proteins such as FN and transforming growth factor-beta 1 (TGF-β1) in HG-treated GMCs via the Nrf2 pathway, as well as exert anti-oxidative stress effects in the kidney tissues of diabetic mice to alleviate renal fibrosis (Chen et al., 2017). Then we speculated whether Fr could activate the downstream anti-oxidative signaling pathway by affecting Cx43 expression. Our results demonstrated that administering Fr to GMCs cultivated in HG reversed the declined in Cx43 protein expression and activated the Nrf2 pathway, which was followed by a decrease in FN and ICAM-1 protein production. However, Fr administration failed to activate the Nrf2 pathway when siRNA was used to interfere with Cx43 expression. The above evidences confirmed that hyperglycemia-induced reduction of Cx43 was essential for the development of metabolic diseases, and up-regulating Cx43 expression might be a potential approach to improve the metabolic class of diabetic diseases. Therefore, we hypothesized that Cx43 might be involved in the regulation of the Nrf2 anti-oxidative pathway by Fr.

Cx43 can regulate cell adhesion, cell migration and other processes through its CT interactions with a variety of signaling and scaffolding proteins, independent of its well-established gap junction communication function (Giepmans, 2006; Hervé et al., 2007). In glioblastoma cells, Cx43 inhibits the activation of the epidermal growth factor signaling pathway by inhibiting AKT hyperphosphorylation through CT linking to AKT (Kuang et al., 2018; Leithe et al., 2018). Another study clarified that isoproterenol decreased cell adhesion in U937 monocytes by inhibiting Cx43 and its downstream PI3K/AKT/NF-κB signaling pathway (Ji et al., 2019). Then, we performed immunofluorescence and IP experiments to investigate whether AKT mediates the activation of the Nrf2 signaling pathway by Cx43. By immunofluorescence detection, we found that Cx43 and AKT co-localized in the cell membrane and cytoplasm of GMCs. The IP experiments results revealed that Cx43 interacted with AKT in normal glucose-cultured GMCs. HG stimulation reduced this interaction while Fr treatment increased it. In addition, we observed both AKT phosphorylation and Nrf2 pathway activation were suppressed when the cells were given MK-2206. Therefore, an important finding in this study was that Fr could increase AKT phosphorylation through upregulation of Cx43, promote the accumulation of Nrf2 in the nucleus and raise the expression of downstream effector proteins, all of which results in antioxidative stress effects.

Moreover, *in vivo* experiments showed that Cx43 expression was downregulated in the kidneys of db/db mice, and these changes were accompanied by a decrease in total SOD activity and an increase in MDA levels in the kidneys and serum, which were reversed by Fr treatment. Fr also raised the expression of the phosphorylated-AKT, Nrf2, SOD1 and HO-1 in the kidneys of db/db mice. Ultimately, Fr alter protein levels of renal inflammatory fibrosis components and alleviate renal insufficiency and renal fibrosis.

## Conclusion

Taken together, the *in vitro* and *in vivo* experiments suggested that Fr promoted AKT phosphorylation and activated the Nrf2/ARE pathway by up-regulating Cx43 expression, thereby decreasing renal oxidative stress levels and ultimately ameliorating the pathological progress of diabetic renal fibrosis. Consequently, we revealed the mechanism through which Fr decreased renal oxidative stress and attenuated diabetic renal fibrosis was associated to the Cx43-AKT-Nrf2 signaling pathway, providing new experimental evidence of the potential clinical application of Fr as a new anti-DN drug.

## Data Availability

The original contributions presented in the study are included in the article/supplementary material, further inquiries can be directed to the corresponding authors.

## Abbreviations

DN, diabetic nephropathy; GMCs, glomerular mesangial cells; Cx43, connexin43; Nrf2, nuclear factor E2-related factor 2; ARE, antioxidant response element; AKT, serine/threonine kinase (B); FN, fibronectin; ICAM-1, intercellular adhesion molecule-1; H2O2, hydrogen peroxide; ROS, reactive oxygen species; HO-1, heme oxygenase 1; SOD, superoxide dismutase; Fr, Fraxin; STZ, streptozocin; TGF-β1, transforming growth factor-β1; ECM, extracellular matrix; KW/BW, kidney/body weight ratio; HbAlc, glycated hemoglobin; GSP, glycated serum protein; BUN, blood urea nitrogen; Cr, serum creatinine; UP, urine protein; MDA, malondialdehyde.
